# Activity of a Novel Anti-Inflammatory Agent F-3,6′-dithiopomalidomide as a Treatment for Traumatic Brain Injury

**DOI:** 10.3390/biomedicines10102449

**Published:** 2022-09-30

**Authors:** Shih Chang Hsueh, Michael T. Scerba, David Tweedie, Daniela Lecca, Dong Seok Kim, Abdul Mannan Baig, Yu Kyung Kim, Inho Hwang, Sun Kim, Warren R. Selman, Barry J. Hoffer, Nigel H. Greig

**Affiliations:** 1 Drug Design & Development Section, Translational Gerontology Branch, Intramural Research Program National Institute on Aging, NIH, Baltimore, MD 21224, USA; 2 AevisBio, Inc., Gaithersburg, MD 20878, USA; 3 Aevis Bio, Inc., Daejeon 34141, Korea; 4 Department of Biological and Biomedical Sciences, Aga Khan University, Karachi 74800, Pakistan; 5 Department of Neurological Surgery, Case Western Reserve University and University Hospitals, Cleveland, OH 44106, USA

**Keywords:** pomalidomide, immunomodulatory imide drugs (IMiDs), neuroinflammation, traumatic brain injury, cereblon

## Abstract

Traumatic brain injury (TBI) is a major risk factor for several neurodegenerative disorders, including Parkinson’s disease (PD) and Alzheimer’s disease (AD). Neuroinflammation is a cause of later secondary cell death following TBI, has the potential to aggravate the initial impact, and provides a therapeutic target, albeit that has failed to translate into clinical trial success. Thalidomide-like compounds have neuroinflammation reduction properties across cellular and animal models of TBI and neurodegenerative disorders. They lower the generation of proinflammatory cytokines, particularly TNF-α which is pivotal in microglial cell activation. Unfortunately, thalidomide-like drugs possess adverse effects in humans before achieving anti-inflammatory drug levels. We developed F-3,6′-dithiopomalidomide (F-3,6′-DP) as a novel thalidomide-like compound to ameliorate inflammation. F-3,6′-DP binds to cereblon but does not efficiently trigger the degradation of the transcription factors (SALL4, Ikaros, and Aiolos) associated with the teratogenic and anti-proliferative responses of thalidomide-like drugs. We utilized a phenotypic drug discovery approach that employed cellular and animal models in the selection and development of F-3,6’-DP. F-3,6′-DP significantly mitigated LPS-induced inflammatory markers in RAW 264.7 cells, and lowered proinflammatory cytokine/chemokine levels in the plasma and brain of rats challenged with systemic LPS. We subsequently examined immunohistochemical, biochemical, and behavioral measures following controlled cortical impact (CCI) in mice, a model of moderate TBI known to induce inflammation. F-3,6′-DP decreased CCI-induced neuroinflammation, neuronal loss, and behavioral deficits when administered after TBI. F-3,6′-DP represents a novel class of thalidomide-like drugs that do not lower classical cereblon-associated transcription factors but retain anti-inflammatory actions and possess efficacy in the treatment of TBI and potentially longer-term neurodegenerative disorders.

## 1. Introduction

Traumatic brain injury (TBI) is a leading cause of death and disability in children and adults. Sixty-nine million individuals worldwide are estimated to be affected by TBI each year [1]. TBI creates damage to the brain by shearing forces, direct contact, or penetration [2,3]. Post-injury impairments, including cognitive, motor, and mood, have been documented after TBI; noteworthy are deficits in gait and locomotion [4]. Approximately 81% of brain injuries are classified as mild TBI, 11% as moderate TBI, and 8% as severe TBI [1]. In addition to the direct injury and functional impairments induced by TBI, accumulating epidemiological data shows that there is a strong link between TBI and the later development of chronic neurodegenerative disorders, such as Parkinson’s disease (PD) and Alzheimer’s disease (AD) [5,6,7,8,9].

Neuroinflammation is an essential component of the secondary sequelae after TBI and is the focus of accumulating research as a potential target for drug development. In pathological states, such as TBI, neuroinflammation can either aggravate or ameliorate brain tissue damage [10,11]. Chronic neuroinflammation after TBI is considered to result in poor outcomes, with further development of several neurodegenerative disorders [11,12,13,14]. The hallmarks of neuroinflammatory responses after TBI are blood-brain barrier (BBB) disruption, edema, the activation and relocation of astrocytes and microglia, the production and release of cytokines and chemokines, and the recruitment of blood-derived leukocytes, such as neutrophil infiltration, into the local area [15,16,17,18,19]. Both clinical and pre-clinical studies have indicated a marked expression of several cytokines after TBI, such as tumor necrosis factor (TNF-α), transforming growth factor-β (TGF-β), and interleukins-1β (IL-1β), IL-6, and IL-10. In addition, there are changes in levels of Keratinocyte Chemoattractant (KC)/human growth-regulated oncogene (GRO) chemokines (KC/GRO, CXCL1), Interferon-gamma (IFN-γ), following downstream signaling of the nuclear factor Kappa-light-chain-enhancer of activated B cells (NF-κB) pathway [13,15,20,21,22,23,24,25]. Among all the mediators influenced after TBI, TNF-α serves as a critical regulator in acute stage neuroinflammation, triggering signaling cascades of pro-inflammatory cytokines [26], and associated with several neurodegenerative disorders, including TBI [27,28], AD [29,30], PD [31,32], multiple sclerosis (MS) [33,34], and amyotrophic lateral sclerosis (ALS) [35,36]. Within minutes to hours after TBI, the mRNA and protein expressions of TNF-α are elevated in patients [37,38] and pre-clinical animal models [39,40], occurring before the presence of other cytokines and enhancing the process of leukocyte infiltration to the local injury site [41]. Previous studies suggest that excessive expression of TNF-α can be detrimental after TBI [28,42,43], and hence, the level and activity of TNF-α need to be regulated, as it may be crucial for long-term recovery [21,28,42].

Thalidomide (α-phthalimidoglutarimide) was a sedative drug developed to replace barbiturate drugs at the end of the 1950s [44] and was later found to be a potent TNF-α inhibitor [45] by inducing TNF-α mRNA degradation [46,47,48]. Because of its adverse teratogenic effects [49,50,51], other structural analogs of thalidomide have been developed to enhance drug efficacy and potentially eliminate adverse effects. In addition to the inhibition of TNF-α expression, thalidomide and its derivatives have also been found to have anti-angiogenic [52,53], anti-proliferative [53,54,55], and anti-inflammatory properties. They have actions inhibiting tumor outgrowth and have been extensively used for the treatment of multiple myeloma, refractory anemia, and Crohn’s disease [56,57,58]. The primary target of thalidomide teratogenicity, as well as anti-cancer actions, is cereblon, a substrate receptor for the ubiquitin E3 ligase complex Cullin-RING ligase 4 (CRL4) [59]. The chemical portion of thalidomide and its derivatives, key to cereblon binding, is the glutarimide ring [60]. This is a flat, planar moiety that can form hydrogen bonds with specific amino acids in the cereblon pocket structure; the phthalimide group on the opposite side plays a supportive role in assisting certain substrates for binding to cereblon, such as Spalt-like transcription Factor 4 (SALL4), zinc finger transcription factors Ikaros (IKZF1), and Aiolos (IKZF3), as well as targets that lack zinc fingers, such as Casein kinase CK-1α, to induce protein degradation through the proteasomal pathway [61].

We used a “phenotypic screening” strategy [62,63] for our novel compounds, which are generated based on the chemical structure of thalidomide and its clinically available analogs, and we selected those that showed anti-inflammatory properties under phenotypic anti-inflammatory screening. We focused on reducing TNF-α and nitrite generation [64]. Pomalidomide is a third-generation thalidomide analog with an amino group at the four-position carbon on the phthaloyl ring of thalidomide [65]. It is an FDA-approved drug used for relapsed/refractory multiple myeloma treatment [65,66] and is found to have more potency in reducing TNF-α and less neurotoxicity compared with thalidomide [67]. It still possesses teratogenicity [68]. However, in addition to its beneficial effect in cancer treatment, pomalidomide serves as a potent agent in anti-inflammation and neuroprotection and has been well documented in many studies. It is noteworthy that pomalidomide can ameliorate neuroinflammation, neuronal loss, and behavioral deficits in several rodent models of TBI [69,70]. 

In this study, we assessed the efficacy of F-3,6′-DP, a novel fluorinated dithio-pomalidomide structural analog [71], in both in vitro and in vivo models. Fluorine has been widely utilized in novel drug synthesis to reduce metabolism [72], and thionation on the pomalidomide backbone could potentially disrupt cereblon-dependent ubiquitination on several neo-substrates, such as SALL4, Aiolos, and Ikaros [73,74], which contribute to the teratogenic and anticancer properties of this class of drug [75,76]. In the current study, we evaluated the cereblon binding of F-3,6′-DP and its actions on neo-substrates, as well as whether F-3,6′-DP can efficiently reduce inflammatory mediators in cellular and rodent models of lipopolysaccharide (LPS)-induced inflammation. Finally, we evaluated its efficacy in mitigating behavioral and histological impairments due to neuroinflammation caused by TBI in rodent models. 

## 2. Methods

### 2.1. Animal Studies

All rodents were housed at 25 °C in a 12 h light/12 h dark cycle and given free access to food and water. All efforts were made to minimize animal suffering and to decrease the number of animals used by integrating the outcome measures from our previous studies and the statistical power analysis. All procedures used in this study were fully approved by the Institutional Animal Care and Use Committee (Intramural Research Program, National Institute on Aging, NIH (protocol No. 331-TGB-2024 and 488-TGB-2022)) and followed the NIH guidelines for research on rats and mice. To avoid confounds because of the potential neuroprotective effects of estrogen, only male animals were used in this ‘first-in-animal’ study of 3,6’-DP.

#### 2.1.1. Synthesis and Analysis of F-3,6′-DP

**Figure 1:**
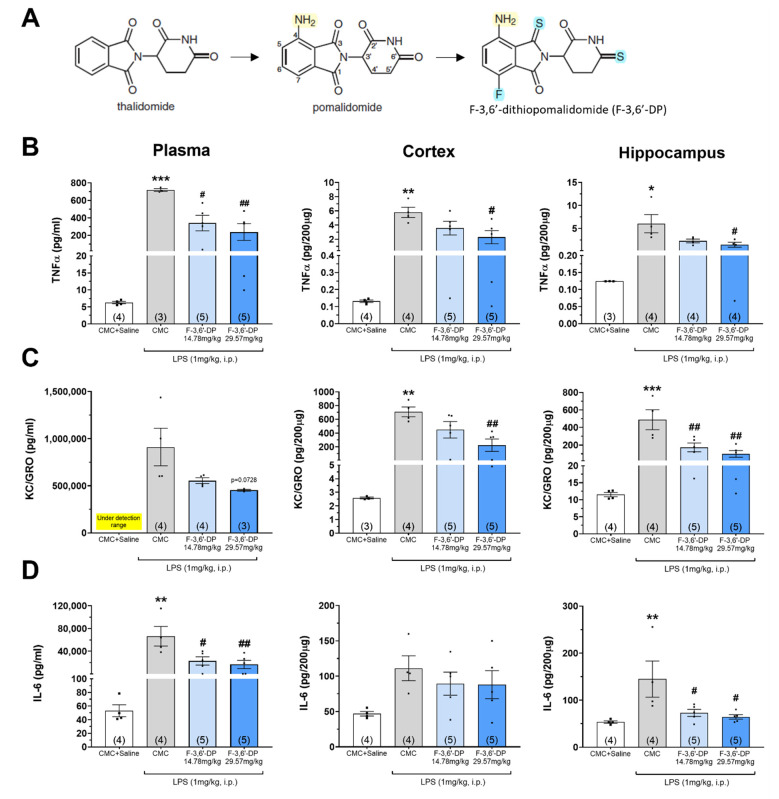
**F-3,6′-DP significantly reduces LPS-induced proinflammatory cytokine (TNF-α, IL-6), chemokine (KC/GRO (CXCL1)) in the plasma, cortex, and hippocampus of the brain.** (**A**) Chemical structures of immunomodulatory drugs (IMiDs), thalidomide, pomalidomide, and F-3,6′-DP. Treatment of the animals with LPS (1 mg/kg, i.p.) markedly elevated levels of proinflammatory TNF-α (**B**), KC/GRO (**C**) and IL-6 (**D**) in plasma and brain. Pretreatment of the animals with F-3,6′-DP (14.78 and 29.57 mg/kg, i.p.) mitigated LPS-induced increases in pro-inflammatory proteins. * *p* < 0.05, ** *p* < 0.01, *** *p* < 0.001 refers to the effects of LPS compared to the control value (CMC + Saline). # *p* < 0.05, ## *p* < 0.01 refers to the effect of drug treatments vs. CMC + LPS. Values are presented as mean ± S.E.M., of n observations (CMC + Saline, n = 4; LPS + CMC, n = 4; LPS + F-3,6′-DP, n = 5 in each dose).

Representative procedure: 7-fluoro-pomalidomide (27 mg, 0.091 mmol, 1.0 eq) and P_4_S_10_-pyridine complex (52 mg, 0.14 mmol, 1.5 eq) were placed into a vial along with a stir bar. After the vessel was sealed and placed under N_2_, 2.5 mL of 1,4-dioxane was injected, and the mixture was stirred and heated to 100 °C. The reaction, initially cloudy yellow, proceeded to a homogenous clear orange solution and finally to a deep red oily mixture. After 20 h, the reaction was stopped and cooled to room temperature, at which point a thick dark oily residue remained at the bottom of the reaction vial. The process from here branched into two parts (A and B) that were ultimately reunited in a final extraction procedure. (A) The red reaction liquor was decanted, evaporated to dryness, redissolved in ethyl acetate, and set aside for later use. (B) The dark oily residue that remained in the original reaction flask was then combined with ethyl acetate and saturated sodium bicarbonate (NaHCO_3_) and stirred gently, at which point the thick oil fully dissolved and partitioned into a red organic layer and a slightly cloudy aqueous layer. These layers were transferred to a separatory funnel and combined with the ethyl acetate material from part A, resulting in a combined red organic layer upon a lower bicarbonate aqueous layer. This initial mixture was not shaken in the traditional manner due to emulsion formation; rather, the aqueous layer was simply drained off. Subsequently, an additional wash with saturated NaHCO_3_ was performed in a shaken manner, followed by washings with water (1×), 0.5M HCl (1×) and finally brine (1×) before being dried over sodium sulfate. The organics were decanted and evaporated to dryness, leaving a dark red-orange residue that was purified by prep-HPLC to yield F-3,6′-DP (Figure 1A) as a bright orange-red powder, 8.6 mg, 29% yield (average of three runs). ^1^H NMR (400 MHz, DMSO-d_6_) δ 12.65 (bs, 1H) 7.9-7.54 (bs, 2H), 7.42 (d, J = 8.3 Hz, 1H), 7.25 (bs, 1H), 5.85-5.40 (bm, 1H*), 3.24-2.78 (bm, 2.4H*), 2.01 (bs, 1H). ^19^F NMR (377) MHz, DMSO-d_6_) δ −127.85 (bm, 1F*). Note that the starred * signals are very broad and difficult to resolve, even after increasing relaxation delays and scans. HRMS: HRMS (MALDI) calculated for [C_13_H_10_FO_2_N_3_S_2_ + Na]^+^ 346.00907, found 346.00931. Elemental analysis: Anal. Calcd for C_13_H_10_FO_2_N_3_S_2_: C, 48.29; H, 3.12; N, 13.00; S, 19.83. Found C, 48.12; H, 3.12; N, 12.97; S, 19.68. These analyses are further supported by X-ray crystallography [71].

Systemic and brain LPS anti-inflammatory studies: Adult male Fischer 344 rats (approx. 150 g weight) were randomly assigned across groups, and then given F-3,6′-DP (14.78 or 29.57 mg/kg, equimolar to 12.5 and 25 mg/kg thalidomide), i.p., suspended in 1% carboxymethyl cellulose (CMC) in normal saline or vehicle 60 min before administration of LPS (1 mg/kg, Sigma, St. Louis, MO, USA, in normal saline, 0.5 mg/mL, or vehicle). Thus, a 100 g animal received 0.2 mL of LPS, i.p. or vehicle. These concentrations of the drug were chosen for the in vivo evaluation of F-3,6′-DP as they are equimolar to, or are less than, doses of thalidomide and analogs that have been verified to be well tolerated in our previous studies and are of translational application to humans. Four hours after LPS injection, the animals were euthanized (see histological analysis methods below), and blood and brain tissue were collected and placed on wet ice. Plasma was quickly separated from blood by centrifuge at 10,000× *g*, 5 min, 4 °C, and brain regions of cortex, hippocampus, and the rest of brain were dissected into separate vials on wet ice and stored at −80 °C. Brain tissues were then sonicated in a TRIS-based lysis buffer (Mesoscale Discovery) with 3× protease/phosphatase inhibitors (Halt™ Protease and Phosphatase Inhibitor Cocktail, Thermo Fisher Scientific). Tissues were then centrifuged at 10,000× *g*, 10 min, 4 °C, and protein concentrations were measured by bicinchoninic acid assay (BCA, Thermo Fisher Scientific). Rat plasma, cerebral cortical, and hippocampal samples were analyzed by multi-proinflammatory cytokine ELISA (V-PLEX Proinflammatory Panel 2 Rat Kit, Mesoscale Discovery) following the manufacturer’s protocol. Plasma levels of KC/GRO (CXCL1) consistently fell outside the linear range of the V-PLEX assay and hence were analyzed by a Rat CINC-1 ELISA kit (abcam, Boston, MA, USA). The protocols and dosages for the experiment of drug treatment effects on the LPS rat model were made based on our previous studies [69].

In Vivo Model of TBI: TBI studies were conducted in 8-week-old male C57/BL6 mice (25–30 g; n = 20), Jackson Laboratory, Bar Harbor, Maine, USA. Mice were randomly assigned to four groups prior to surgery: (1) CMC + Sham, (2) CMC + TBI, (3) F-3,6′-DP (Low dose (LD), 14.78 mg/kg, prepared in 1% CMC in PBS) + TBI, or (4) F-3,6′-DP (High dose (HD), 29.57 mg/kg, prepared in 1% CMC in PBS) + TBI. All groups were evaluated for the effects of F-3,6′-DP on TBI. Mice were assessed for gait function, motor coordination, and balance function. The animals were subsequently evaluated for cellular changes using histology and immunohistochemistry after sacrifice.

**Figure 2:**
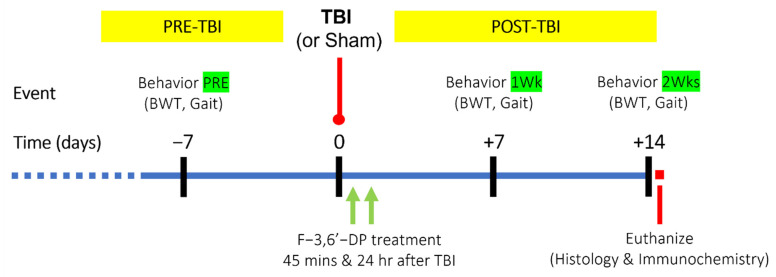
**Timeline of TBI with F-3,6′-DP treatment study design.** Mice were first evaluated for their baseline motor coordination/balance, and gait functions by the beam walking test (BWT), and DigiGait analysis one week before TBI injury (PRE). All mice received the first injection of F-3,6′-DP or CMC vehicle at 45 min after TBI and a 2nd injection on the following day. Behavioral tests were performed on 7 and 14 days after TBI, and thereafter, mice were euthanized for assessment of histology and immunochemistry.

For TBI, mice were anesthetized with 2.5% tribromoethanol (Avertin: 250 mg/kg; Sigma, St. Louis, MO, USA), placed in a mouse stereotaxic frame (Kopf Instruments, Tujunga, CA, USA), and fixed with ear bars and an incisor bar. Under sterile conditions, the skin was retracted, and a 5-mm square craniectomy was placed over the right motor cortex at the posterior corner between the bregma and sagittal sutures. The skull was carefully removed with a drill to avoid damaging the dura underneath. The craniectomy was carried out without having the temporalis muscle impaired. The CCI device, Impact One (Leica Biosystems Inc., Buffalo Grove, IL, USA), consists of an electromagnetic impactor that allows for the alteration of injury severity by controlling contact velocity and the depth of cortical deformation independently. Prior to impact, the tip of the 3-mm flat impactor was angled and kept perpendicular to the exposed cortical surface. The contact velocity was set at 5 m/s, dwell time was set at 0.2 s, and deformation depth was set at 2 mm to produce moderate TBI. After the impact, we used sterile cotton-tipped applicators to clean the area around the injury site and then closed the wound with surgical sutures (Ethicon Inc., Somerville, NJ, USA). Sham animals were anesthetized and followed the same procedure as TBI mice without impact. During surgery and recovery, the core body temperature of all mice was maintained at 36–37 °C using either a heat pad or a heated chamber. Mice were returned to their home cage after awakening from anesthesia. Mice were then given F-3,6′-DP (14.78 or 29.57 mg/kg in 0.1 mL/10 g body weight) or CMC vehicle by i.p. injection, with the first injection administered 45 min after injury and the second injection on the next day (Figure 2).

##### Beam Walk Test

TBI-induced impairments in motor coordination were evaluated using a beam walk test (BWT). Mice have an intrinsic tendency to stay in a darkened enclosed environment compared to an open illuminated field. Each mouse was placed in the darkened goal box for a 2 min habituation and was then moved to the other (light) end of the beam to start the trial. Time spent and the number of footfalls occurring during the crossing of the beam were recorded at baseline (PRE), and 1 and 2 weeks after TBI, with the caveat that total time was not to exceed 30 s. The dimensions of the beam were 1.2 cm (width) × 91 cm (length). The time taken for each animal to traverse the beam to reach the dark goal box, and the number of ipsilateral and contralateral foot falls were documented. Five trials were recorded for each animal before CCI and 1 and 2-weeks after CCI. The mean times to traverse the beam were calculated, and a plot was generated to evaluate the treatment effects on beam walk times and footfalls; these times were then used for analysis. 

##### Gait Analysis

A DigiGait treadmill system was used to analyze gait parameters per the manufacturer’s protocol (Mouse Specifics, Inc., Framingham, MA, USA) at baseline (PRE), 7 days (1 Wk), and 14 days (2 Wks) post injury. At each time point, each animal was moved to the test chamber and allowed to acclimate for 2 min to the new environment, while software was set up and bumpers adjusted to maintain the animal in the field of view. The treadmill was initially started at 5 cm/s and the animal was allowed to run for 1 min, then given a break for 1 min. The speed was gradually increased to the testing speed of 15–20 cm/s at which time recording was initiated. Once 3–5 s of constant stepping was captured, the animal was then returned to its home cage.

Only trials in which the camera recorded all gait parameters were included in the analysis. The videos were analyzed with DigiGait software to evaluate stride duration, length, paw angle, stance, etc., and were evaluated for all four limbs (Mouse Specifics, Inc.). Parameters included brake time (duration time of the braking phase of initial paw contact to maximum paw contact, commencing after the swing phase), percent of brake phase (percent of the total stride duration that the paw is in the braking phase), propel time (duration time of the propulsion phase, maximum paw contact to just before the swing phase), percent of propel phase (percent of the total stride duration that the paw is in the propulsion phase), and paw angle variability (the standard deviation of the paw angle for the set of strides recorded). All assessments were made by an investigator blinded to the treatment group.

##### Histological Analysis

Fixation and sectioning: Animals were anesthetized with 2.5% tribromoethanol and Avertin (Sigma, St. Louis, MO, USA) and perfused transcardially with 0.9% saline and 4% PFA in 0.1 M phosphate buffer (PB, pH 7.2). Brains were removed and post-fixed for 1 day in 4% PFA and sequentially transferred to 20% and 30% sucrose in 0.1 M PB until the brain sank. The brains were cut into 25 μm sections on a cryostat (Leica Biosystems Inc., Buffalo Grove, IL, USA). Every seventh section was selected from a region spanning the striatum to the hippocampus.

Quantification of brain lesion and lateral ventricle size in TBI animals: One set of post-TBI 2-week brain sections (25 μm) were mounted on slides. The sections were then stained in 10% Giemsa KH_2_PO_4_ buffered solution (pH 4.5) for 30 min at 40 °C. After a brief rinse, the slides were de-stained, differentiated, and dehydrated in absolute ethanol. Thereafter, the sections were cleared in xylene and then cover-slipped. Slides were scanned in an All-in-One Fluorescence Microscope BZ-X710 (Keyence Corporation of America, Itasca, IL, USA), and brain image areas were quantified using ImageJ 1.52q software (National Institutes of Health, Bethesda, MD, USA). The calculation formula for contusion volume size and lateral ventricle size was as follows: Ʃ (area of contralateral hemisphere—area of ipsilateral hemisphere)/Ʃ area of contralateral hemisphere ×100; Ʃ area of ipsilateral lateral ventricle/Ʃ area of contralateral lateral ventricle. There were 9 brain sections from each mouse counted, with regions starting from bregma 0.86 mm to −1.46 mm.

##### Immunofluorescence

Four brain sections per mouse were incubated with blocking buffer (4% Bovine Serum Albumin, Sigma, St. Louis, MO, USA) for 1 h. A series of primary antibodies was prepared in the blocking buffer, and the sections were incubated in this solution overnight. The antibodies used were goat anti-GFAP (Glial Fibrillary Acidic Protein) (1:500; Abcam, Cambridge, MA, USA), or rabbit anti-Iba1 (Ionized calcium binding adaptor molecule 1) (1:500; FUJIFILM Wako Pure Chemical Corporation, Richmond, VA, USA). After incubation with primary antibody, the sections were washed and incubated for 3 h at room temperature in diluted secondary antibody prepared with blocking solution (secondary antibody conjugated with Alexa 488 or 555 (1:500; Thermo Fisher Scientific, Waltham, MA, USA). The sections were then washed with 0.1 M PB (pH 7.2), mounted with Antifade Mounting Medium with DAPI (Vector, Burlingame, CA, USA), and cover-slipped. A series of 4 images per mouse brain were taken using a Laser Scanning Microscope (Zeiss 710, Oberkochen, Germany). The cell numbers of each image were counted using ImageJ 1.52q software (National Institutes of Health, Bethesda, MD, USA). Controls consisted of omission of primary antibody, and observers were blinded to treatment groups. (Immunofluorescence analysis and quantification): Iba1-positive microglia and GFAP-positive astrocytes were identified with an ×40 oil magnification objective. For each mouse, four to six cortex fields were captured from both the ipsilateral and contralateral hemispheres. The immunoreactive (IR) cell numbers of microglia and the total area of GFAP IR in each field were quantified using the NIH software ImageJ 1.52q. The observers were blinded to the treatment group.

In relation to Iba1 immunostaining, microglial cells were subclassified by morphological subtypes, in line with prior studies, as microglial morphology is considered highly representative of their functional state. These subtypes included ramified and intermediate-type microglial cells, as well as amoeboid and round subtypes. Morphometric parameters were analyzed using MotiQ, fully automated analysis software. MotiQ was developed as an ImageJ plugin in Java and is publicly available (https://github.com/hansenjn/MotiQ, accessed on 27 July 2022). The microglial ramification index is the ratio of the cell surface area to the surface area of a perfect sphere with the same volume as the analyzed cell. The ramification index is a unit-free parameter for the complexity of cellular shapes. A ramification index of 1 corresponds to a perfectly round cell without processes. The more the cell differs from a perfectly round shape, i.e., the more branches the cell possesses, the higher its 3D ramification index. All segmentation and quantification were performed on the maximum intensity projections of 3D image data.

#### 2.1.2. Statistical Analysis

For behavioral data, a two-way repeated measures analysis of variance (ANOVA) was used to assess both groups and time factors. Multiple within-subject comparisons were evaluated with Dunnett’s test when the main effect of time was significant. For analysis of multi-proinflammatory cytokines ELISA, contusion volume size, and lateral ventricle size, a one-factor analysis repeated measures ANOVA was used to compare the 4 groups of data followed by a Dunnett’s test on 2-weeks post lesion. The data were analyzed using GraphPad Prizm 7 (San Diego, CA, USA) with the significance level set at *p* < 0.05 for each assessment. All data are presented as the average ± standard error of the mean (SEM).

### 2.2. Cellular Studies

#### 2.2.1. Cereblon Binding and Neo-Substrate Assays

A bead-based AlphaScreen technology was adopted for cereblon binding, with minimal modifications from the manufacturer’s protocol (BPS Bioscience catalog no. 79770). F-3,6′-DP or pomalidomide was incubated with reaction mixtures including cereblon/DNA damage-binding protein 1−Cullin 4a−ring-box protein 1 complex (CRBN/DDB1−CUL4A−Rbx1, 12.5 ng) and bromodomain-containing protein 3 (BRD3) (6.25 ng) in an Optiplate 384-well plate (PerkinElmer catalog no. 6007290). After 30 min of incubation with shaking at room temperature, AlphaLISA anti-FLAG Acceptor and Alpha Glutathione Donor beads (PerkinElmer) were sequentially added and then incubated for 1 h at room temperature for each of the added chemicals. Alpha counts were thereafter read on a Synergy Neo2 (BioTek) for the analysis. The relative activity of the alpha signal was calculated after subtraction of the “blank value” from all readings, and the value of the vehicle group was then set as 100%.

The effect of F-3,6′-DP activity on neo-substrates was evaluated in both MM1.S (myeloma) and Tera-1 cell lines. Specifically, MM1.S cells were obtained from ATCC (Manassas, VA, USA), grown in RPMI media supplemented with 10% FBS, penicillin 100 U/mL, and streptomycin 100 mg/mL, and maintained at 37 °C and 5% CO_2_. MM1.S cells were treated with 1 μM of pomalidomide or F-3,6′-DP for 24 h; thereafter, the cell lysates were prepared for Western blot analysis, as described previously [74]. Tera-1 cell lines were obtained from Korean Cell Line Bank (catalog no. 30105; Seoul, Korea) and grown in Dulbecco’s modified Eagle’s medium (DMEM), supplemented with 10% FBS, penicillin 100 U/mL and streptomycin 100 μg/mL, and maintained at 37 °C and 5% CO_2_. Tera-1 cells were treated with pomalidomide or F-3,6′-DP (0.01, 0.1 and 1 μM) for 4 h, and their cell lysates were prepared for the Western blot analysis, as described previously [77].

For Western blot analysis, total proteins were extracted using RIPA buffer (ThermoFisher Scientific, Waltham, MA, USA) containing Halt Protease Inhibitor Cocktail (ThermoFisher Scientific). Thereafter, the proteins were separated by gel electrophoresis and then transferred to polyvinylidene difluoride (PVDF) membranes (ThermoFisher Scientific), as described previously [74]. The following primary antibodies were used: (i) anti-Ikaros antibody (catalog no. 9034; 1:1000; Cell Signaling Technology, Danvers, MA), (ii) anti-Aiolos antibody (catalog no. 15103; 1:1000; Cell Signaling Technology), (iii) anti-SALL4 antibody (catalog no. ab29112; 1:1000; Abcam, UK), and (iv) anti-GAPDH antibody (catalog no. ab8245; 1:5000; Abcam, UK). After incubation at 4 °C overnight, the following HRP-conjugated secondary antibodies were used: (i) goat anti-rabbit IgG (ThermoFisher Scientific) for Ikaros and Aiolos, and (ii) goat anti-mouse IgG (ThermoFisher Scientific) for SALL4 and GAPDH. GAPDH, a protein that is generally expressed in all eukaryotic cells, was used as an internal control against which the other protein expression levels were compared. Antigen−antibody complexes were detected using enhanced chemiluminescence (ThermoFisher Scientific, iBright CL1500).

#### 2.2.2. Cellular Studies in RAW Cells

F-3,6′-DP activity in LPS-stimulated RAW 264.7 cells. Mouse RAW 264.7 cells, originally acquired from ATCC (Manassas, VA), were grown in DMEM supplemented with 10% fetal calf serum (FCS), penicillin 100 U/mL and streptomycin 100 μg/mL, and were maintained at 37 °C and 5% CO_2_. The cells were grown in accordance with ATCC guidelines, as previously described [74]. On the day of the study, RAW 264.7 cells were challenged with LPS (Sigma, St. Louis, MO: serotype 055:B5) at a final concentration of 60 ng/mL. This LPS concentration routinely induces a submaximal rise in both TNF-α and nitrite levels without a loss of cell viability. Such a submaximal rise is useful for assessing whether the addition of an experimental drug can either lower or further elevate the levels of TNF-α and nitrite. In a drug pretreatment paradigm, either F-3,6′-DP (0.6–60 μM) or vehicle (Veh), n = 3–4, was administered 60 min prior to LPS challenge. At 24 h following the addition of LPS, conditioned media was harvested, and both secreted TNF-α protein (ELISA MAX™ Deluxe Set Mouse TNF-α, catalog no. 430904, BioLegend, San Diego, CA, USA) and nitrite levels (Fluorometric Assay Kit, Abnova, catalog no. KA1344, Walnut, CAUSA) were quantified as recommended by the manufacturers. Fresh media were replaced in the wells, and cell viability was thereafter evaluated with a CellTiter 96 Aqueous One Solution Cell Proliferation Assay (Promega, Madison, WI, USA). For cell culture studies, F-3,6′-DP was prepared immediately prior to use in 100% DMSO and then added to cell culture media at a dilution of greater than 200-fold to provide the desired concentrations; control-/veh-treated cells were subjected to the exact same procedure, but without the addition of F-3,6′-DP.

### 2.3. Docking Pockets and Predictions of F-3,6′-DP and Thalidomide Structural Analog Interactions with Cereblon

Evaluations of potential docking pockets and docking predictions for S enantiomeric forms of F-3,6′-DP, thalidomide and pomalidomide, on the structure of cereblon were analyzed. Prospective drug docking pockets were initially determined to support the computation of the pharmacophore engagement by the molecules using automated software [78]. This was followed by a cavity-based blind drug docking prediction utility [79] to evaluate the attributes of the drug docking prediction of these IMiDs. The drug docking pockets and the binding differences between F-3,6′-DP, thalidomide and pomalidomide in cereblon were determined for the best scoring attributes of these chemical agents. Briefly, the crystal structure of human cereblon in complex with DDB1 and lenalidomide (4TZ4: https//www.rcsb.org/structure/4TZ4, accessed on 27 July 2022) was downloaded in PDB format from the PDB database. Chain C (human cereblon) was isolated from the rest of the crystal structure complex and was prepared for docking predictions for the S enantiomeric forms of thalidomide, pomalidomide, and F-3,6′-DP. This enantiomer was selected because prior X-ray crystallographic studies have indicated that the S rather than R enantiomeric form of classic thalidomide-like drugs more optimally binds cereblon [60,61], albeit molecular modeling computational data does not necessarily simulate or fall in line with all empirical data from prior X-ray crystallographic studies. The automated server at Playmolecule, which uses a software DeepSite [78,79] to determine the core binding sites, was used to evaluate potential interactions between F-3,6′-DP, thalidomide and pomalidomide with human cereblon. The S chiral forms of thalidomide and pomalidomide were downloaded and evaluated as this enantiomeric form, as noted above, is reported to have more potent cereblon binding [60,61,75,76]. Automated docking software [80] was used to investigate potential similarities and differences in the pharmacophore pockets engaged by the three drugs. Briefly, two files were uploaded that included the C-chain (human cereblon) of PDB ID 4TZ4 without lenalidomide and damaged DNA binding protein 1 (DDB1) and the drugs individually in their PDB formats to the Docking server [80]. For docking pocket predictions, the results appear as the number of preferential pockets determined by their relevant scores. The results of docking were collected with individual Vina scores, cavity sizes, docking centers, poses, and sizes of predicted cavities for the drugs noted above. The resulting drug-cereblon complexes were visualized using the drug discovery studio visualizer software BIOVIA [78]. The top binding modes of F-3,6′-DP, pomalidomide and thalidomide with Vina scores with their principal binding cavities were selected for comparison of the binding pose of the drugs and the pockets occupied by them.

## 3. Results

### 3.1. Animal Studies

#### 3.1.1. F-3,6′-DP Reduces the Expression of Pro-Inflammatory Cytokines, TNF-α, IL-6 and Chemokine KC/GRO (CXCL1) Induced by LPS in Rats

To evaluate the anti-inflammation activities of F-3,6′-DP in vivo, we utilized the LPS rat model as a screening platform. Systemic treatment with LPS induced significant amounts of TNF-α, KC/GRO (CXCL1), and IL-6 in the plasma, cortex, and hippocampus of the brain (Figure 1). Prior systemic administration of F-3,6′-DP (29.57 mg/kg i.p.) significantly reduced the level of TNF-α in both plasma and brain (Figure 1B), KC/GRO in brain (Figure 1C), and IL-6 in plasma, and hippocampus (Figure 1D). A drug treatment trend in lowering KC/GRO in plasma was additionally evident (F-3,6′-DP *p* = 0.081, vs. CMC + LPS (ANOVA), and F-3,6′-DP 29.57 mg/kg: *p* = 0.0728, vs. CMC + LPS (Dunnett’s test) (Figure 1C).

#### 3.1.2. F-3,6′-DP Ameliorates Behavioral Deficits and Contusion Volume Produced by TBI

To further assess whether the anti-inflammatory properties in the LPS rat model are of benefit to TBI, the ability of F-3,6′-DP to ameliorate TBI-induced impairments was measured in mice. As shown in Figure 2, mice received a controlled cortical impact to mimic TBI at day 0, and behavioral assessments were conducted at 1 week before TBI (day −7), 1 and 2 weeks after TBI (day +7, +14). Either F-3,6′-DP or vehicle (CMC) was given to mice at 45 min and 24 h after TBI, and mice were euthanized at 2 weeks after TBI for histology and immunostaining. 

**Figure 3:**
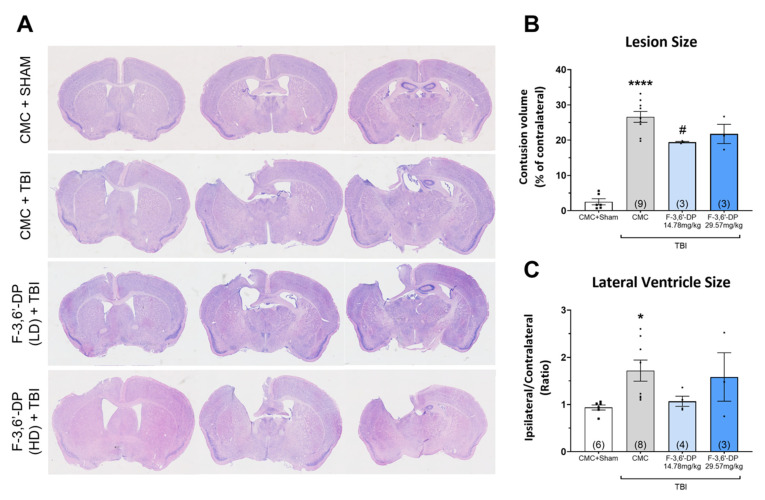
**F-3,6′-DP treatment reduces contusion volume after TBI.** Representative Giemsa-stained coronal brain sections of the TBI-induced cavity in Sham (control without TBI), CMC + TBI, low dose (LD) (14.78 mg/kg) and high dose (HD) (29.57 mg/kg) treatments of F-3,6′-DP in TBI mice at 2 weeks post-TBI (**A**). TBI significantly induced tissue loss in the cortex region of the brain (**B**) and enlargement of the lateral ventricle size (**C**). A significant reduction in lesion size was observed in the low dose (14.78 mg/kg) treated group of F-3,6′-DP after TBI (**B**). * *p* < 0.05, **** *p* < 0.0001 refers to the effects of TBI compared to the control value (CMC + Sham). # *p* < 0.05 refers to the effect of drug treatments vs. CMC + TBI. Values are presented as mean ± S.E.M. of n observations (CMC + Sham, n = 6; CMC + TBI, n = 9; F-3,6′-DP (LD) + TBI, n = 4; F-3,6′-DP (HD) + TBI, n = 3).

Histological staining was performed to evaluate brain tissue loss and lateral ventricle size enlargement induced by TBI. The contusion volume is shown as a percentage of contralateral volume for each group, and the lateral ventricle size is presented as a ratio of ipsilateral versus contralateral size. After TBI, there was a significant tissue loss observed in the ipsilateral cortex (Figure 3A), compared with the sham group (*p* < 0.0001) (Figure 3B), and there was a significant reduction of the lesion size in the F-3,6′-DP low dose treatment group (14.78 mg/kg) compared to the vehicle (CMC) group (*p* < 0.05) (Figure 3B).

We also measured the size of the lateral ventricle as an index of changes in intracranial cerebrospinal fluid (CSF) in the mouse brain after TBI. The enlargement of ventricle size is a typical clinical marker after TBI [81]. We observed a significant increase in the ipsilateral ventricle size after TBI (*p* < 0.05) (Figure 3C). The low dose F-3,6′-DP treatment group had a trend in reducing ventricle size enlargement caused by TBI (*p* = 0.0783, as compared with the CMC + TBI group).

#### 3.1.3. F-3,6′-DP Attenuates Gait Impairments Caused by TBI

**Figure 4:**
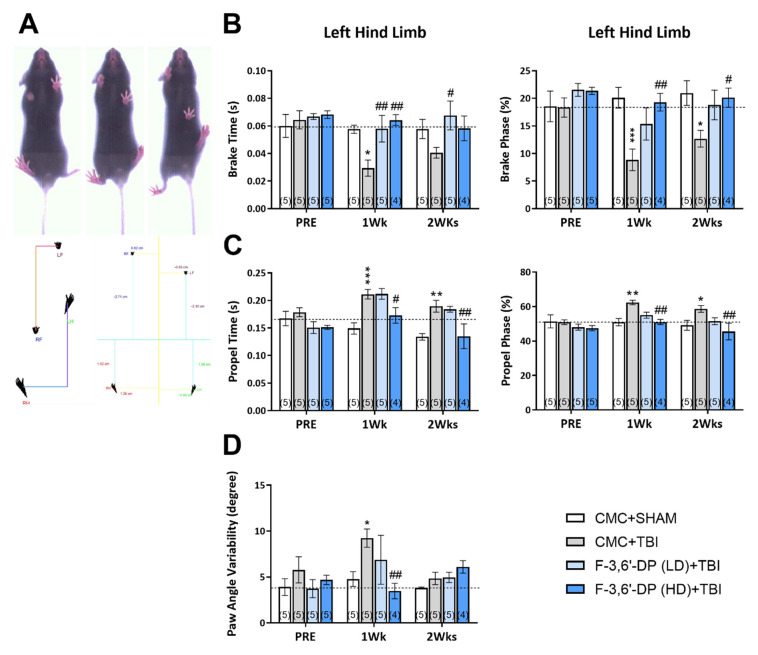
**F-3,6′-DP treatment improved gait functional recovery, as revealed by the DiGi gait assessment.** Gait function was measured using the DiGi gait system (Mouse Specifics, Inc.) (**A**). TBI changed the duration of the braking phase (initial paw contact to maximum paw contact) (**B**). Propulsion phase (maximum paw contact just before the swing phase) (**C**). Percent of the total stride duration that the paw is in the braking phase (**B**). Propulsion phase (**C**) and variability of the paw angle (**D**). TBI-induced gait deficits were reduced by F-3,6′-DP treatment (14.78 and 29.57 mg/kg, i.p.). * *p* < 0.05, ** *p* < 0.01, *** *p* < 0.001 refers to the effects of TBI compared to the control value (CMC + Sham). # *p* < 0.05, ## *p* < 0.01 refers to the effect of drug treatments vs. CMC + TBI. Values are presented as mean ± S.E.M., of n observations (CMC + Sham, n = 5; CMC + TBI, n = 5; F-3,6′-DP (LD) + TBI, n = 5; F-3,6′-DP (HD) + TBI, n = 4).

To determine whether F-3,6′-DP-mediated mitigation in proinflammatory activity and the brain histology noted above are behaviorally relevant, motor parameters were measured before and after cerebral injury in mice with F-3,6′-DP or vehicle post-treatment (Figure 2). DigiGait analysis was performed to measure gait function in TBI and sham mice (Figure 4A). As demonstrated in Figure 4, there were no significant differences in the groups before injury (PRE). TBI significantly decreased the duration of the initial paw contact to maximum paw contact (brake time) and the percent of the total duration that the paw was in the brake phase at 1 and 2 weeks after injury (* *p* < 0.05, *** *p* < 0.001, compared to the CMC + sham group) (Figure 4B). TBI mice treated with F-3,6′-DP (low dose, LD, 14.78 mg/kg) significantly reduced the deficit in brake time at 1 and 2 weeks after TBI (# *p* < 0.05, ## *p* < 0.01, compared with the CMC + TBI group), and the impairment of brake phase was ameliorated in the F-3,6′-DP high dose (HD) treatment group (29.57 mg/kg) (# *p* < 0.05, ## *p* < 0.01, compared with the CMC + TBI group). The duration of the maximum paw contact to just before the paw left the belt (propel time) and the percent of total duration that the paw was in the propulsion phase were significantly elevated by TBI (** *p* < 0.01, *** *p* < 0.001, compared with the CMC + sham group) (Figure 4C), and treatment with F-3,6′-DP (high dose (HD), 29.57 mg/kg) significantly mitigated the elevation in the propel time and % of propel phase elevated by TBI (# *p* < 0.05, ## *p* < 0.01, compared to the CMC + TBI group). The deviation of the paw angle was significantly increased at 1 week after TBI (* *p* < 0.05, compared with the CMC + sham group) and F-3,6′-DP (high dose (HD), 29.57 mg/kg) significantly alleviated this impairment induced by TBI (## *p* < 0.01, compared with the CMC + TBI group) (Figure 4D).

**Figure 5:**
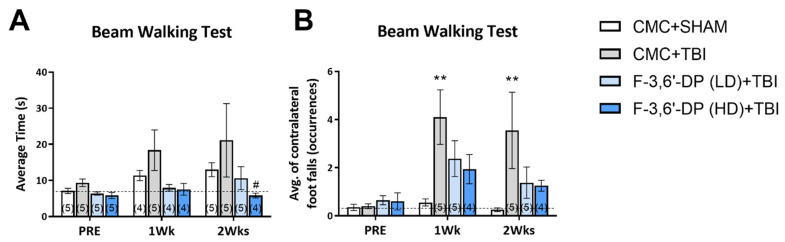
**F-3,6′-DP treatment improved motor coordination and balance function, as revealed by the beam walking test.** Mice challenged with TBI spent more time traversing the beam (**A**) and had markedly increased contralateral foot falls during the test (**B**). F-3,6′-DP treatment (14.78 and 29.57 mg/kg, i.p.) ameliorated the deficits caused by TBI. ** *p* < 0.01 refers to the effects of TBI compared to the control value (CMC + Sham). # *p* < 0.05 refers to the effect of drug treatments vs. CMC + TBI. Values are presented as mean ± S.E.M., of n observations (CMC + Sham, n = 5; CMC + TBI, n = 5; F-3,6′-DP (LD) + TBI, n = 5; F-3,6′-DP (HD) + TBI, n = 4).

The beam walking test was used to measure motor coordination in TBI and sham mice and included two parameters: average transit time (Figure 5A) and number of contralateral foot falls (Figure 5B) while traversing the beam. The average beam transit time increased after TBI, but it did not reach a significant difference (Figure 5A). The number of contralateral foot falls significantly increased after TBI (** *p* < 0.01, compared with the CMC + sham group, Figure 5B). Although we observed a clear reduction trend in both parameters in F-3,6′-DP treated groups, only the average time of the F-3,6′-DP high dose (HD, 29.57 mg/kg) treatment group showed a significant difference (# *p* < 0.05, compared with the CMC + TBI group) (Figure 5A).

#### 3.1.4. F-3,6′-DP Treatment Reduced the Activation of Microglia and Astrocytes Induced by TBI

**Figure 6:**
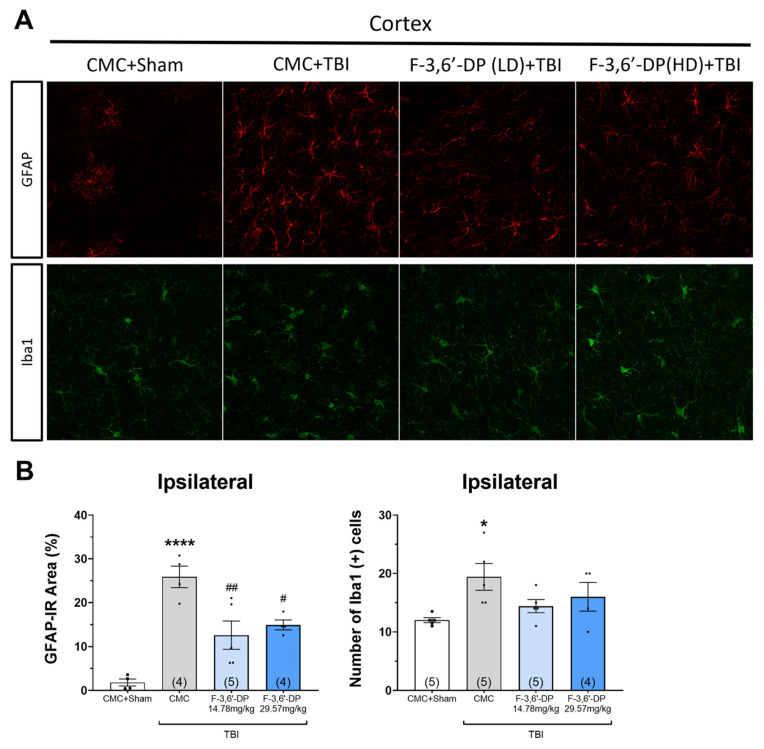
**Post-injury treatment with F-3,6′-DP decreased GFAP-positive astrocytes 2 weeks after TBI.** Immunofluorescence of glial fibrillary acidic protein (GFAP) and Ionized calcium binding adaptor molecule 1 (Iba1) in cortical brain sections. GFAP, a marker for astrocytes, is shown in red. Iba1, a marker of microglia, is shown in green (**A**). TBI injury significantly increased the number of astrocytes and microglia in the ipsilateral cortex. Treatment with F-3,6′-DP significantly reduced the amount of astrocytic elevation caused by TBI (**B**). * *p* < 0.05, **** *p* < 0.0001 refers to the effects of TBI compared to the control value (CMC + Sham). # *p* < 0.05, ## *p* < 0.01 refers to the effect of drug treatments vs. CMC + TBI. Values are presented as mean ± S.E.M., of n observations (CMC + Sham, n = 5; CMC + TBI, n = 5; F-3,6′-DP (LD) + TBI, n = 5; F-3,6′-DP (HD) + TBI, n = 4).

To determine the cellular markers of neuroinflammation in the brain after TBI, the number of astrocytes was measured by GFAP immunostaining in the cortex, and microglia were stained with Iba1. After TBI, we observed significant induction of the number of astrocytes and microglia (* *p* < 0.05, **** *p* < 0.0001, compared with the CMC + sham group) (Figure 6A,B). Treatment with F-3,6′-DP (14.78, 29.57 mg/kg) significantly decreased the number of astrocytes elevated by TBI (# *p* < 0.05, ## *p* < 0.01, compared with the CMC + TBI group) (Figure 6A,B).

**Figure 7:**
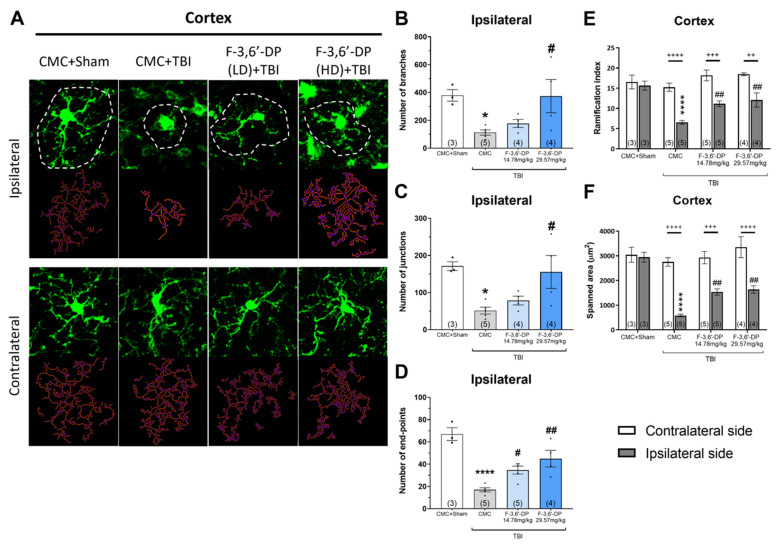
**The effect of F-3,6′-DP treatment 2 weeks after TBI on microglial morphology in the cortex.** Maximum intensity projections of confocal z-stack Iba1 images (green) illustrating the microglial morphology in cortical sections and cell skeleton (red) of representative CMC + Sham, CMC + TBI, F-3,6′-DP(LD) + TBI and F-3,6′-DP(HD) + TBI microglial cells highlighted above (**A**). Quantitative analysis of the number of cell branches (**B**), cell process junctions (**C**), cell process end-points (**D**), ramification index (**E**), cell scanned area, (**F**) of microglia in each group of animals. TBI specifically induced morphological changes in microglia in the ipsilateral cortex (**B**–**F**), and F-3,6′-DP significantly attenuated the effects of TBI. One data point represents the animal mean (out of 18–24 cells per animal). * *p* < 0.05, **** *p* < 0.0001 refers to the effects of TBI compared to the control value (CMC + Sham). # *p* < 0.05, ## *p* < 0.01 refers to the effect of drug treatments vs. CMC + TBI. ++ *p* < 0.01, +++ *p* < 0.001, ++++ *p* < 0.0001 refers to the differences between the ipsilateral and contralateral sides. Values are presented as the mean ± S.E.M. of n observations (CMC + Sham, n = 3; CMC + TBI, n = 5; F-3,6′-DP (LD) + TBI, n = 5; F-3,6′-DP (HD) + TBI, n = 4).

The morphology of microglia indicates different states of activation. The ramified form, with smaller somata and wider process extensions, is considered the “resting” condition of microglia. In this state, the cells possess a “surveying” role in their microenvironment and regulate the neuronal cell’s function. After neuronal injury, microglia transform into an amoeboid morphology by retracting processes and extending the protrusions to become an “active” form [82]. We assessed the microglial morphology by quantifying the images of Iba1-positive cells using the MotiQ plugin under FIJI software. We observed that TBI dramatically changed the morphology of microglia from a ramified form to an amoeboid form in the ipsilateral cortex, without affecting the microglia on the contralateral side (Figure 7A). The number of branches (Figure 7B), junctions (Figure 7C), and endpoints (Figure 7D) of microglial processes were significantly decreased by TBI (* *p* < 0.05, **** *p* < 0.0001, compared with the CMC + sham group) and F-3,6′-DP (14.78, 29.57 mg/kg) significantly reversed these morphological changes in microglia caused by TBI (# *p* < 0.05, ## *p* < 0.01, compared with the CMC + TBI group) (Figure 7B–D). We also used the ramification index, the ratio of surface area to volume, and the spanned area to evaluate the morphology of the microglia. Unilateral impact in the cortex reduces the ramification index and spanned area of microglia, specifically within the ipsilateral side, without influencing the contralateral side (**** *p* < 0.0001, compared to the CMC + sham group) (Figure 7E,F). Treatment with F-3,6′-DP significantly ameliorated the morphological alterations of microglia induced by TBI (## *p* < 0.01, compared with the CMC + TBI group) (Figure 7E,F).

### 3.2. Results: Cellular Studies

#### Cereblon Binding and Neo-Substrate Assays

**Figure 8:**
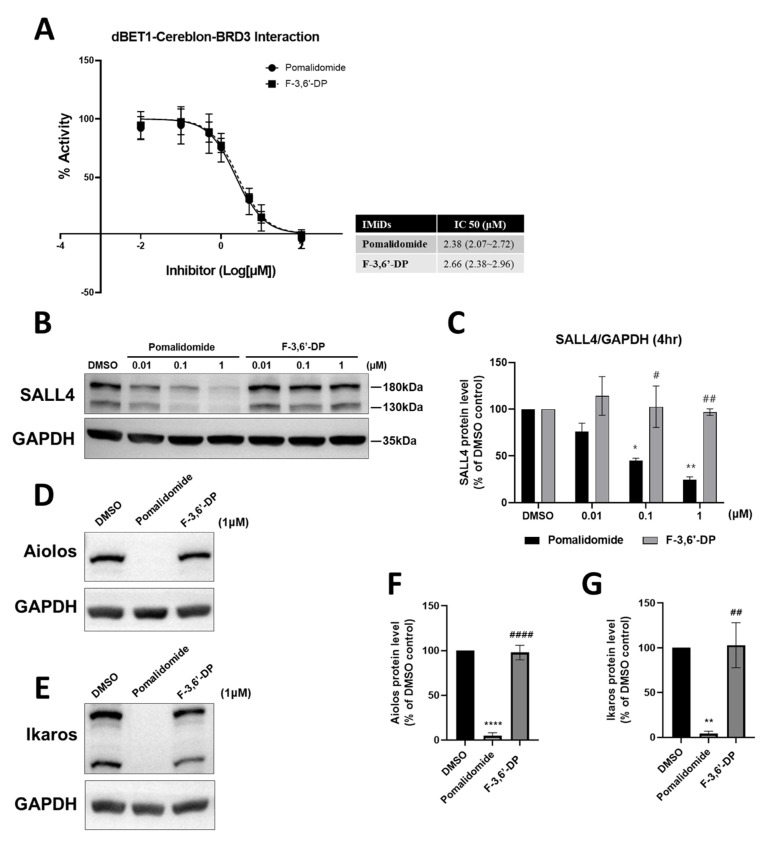
**F-3,6′-DP potently binds Cereblon but does not trigger degradation of the classical neo-substrates SALL4, Ikaros, and Aiolos**. (**A**) A concentration-dependent evaluation of binding between IMiDs and cereblon by FRET assay provided an IC_50_ value of pomalidomide (Pom) and F-3,6′-DP, of 2.38 μM 2.66 μM respectively (N.B., the concentration-dependent curves extensively overlay each other). The thalidomide analog-mediated degradation of neo-substrates, SALL (**B**,**C**), Aiolos (**D**,**F**), and Ikaros (**E**,**G**) was evaluated by Western blotting, and the relative expression level of each neo-substrate was quantified.*, *p* < 0.05, **, *p* < 0.01 ****, *p* < 0.0001 vs. control (Con) value; #, *p* < 0.05, ##, *p* < 0.01, ####, *p* < 0.0001 vs. pomalidomide value (Tukey’s multiple comparisons test).

The binding of pomalidomide and F-3,6′-DP to cereblon was examined using a cereblon/BRD3 binding FRET assay (Figure 8). A concentration-dependent evaluation of binding between pomalidomide and cereblon provided an IC_50_ value of 2.38 μM, whereas the IC_50_ of F-3,6′-DP was 2.66 μM; indicating that both agents potently bind to cereblon (with the concentration-dependent curves largely superimposing one another). The thalidomide analog-mediated degradation of SALL4 in human Tera-1 cells (0.01, 0.1, 1 μM of pomalidomide and F-3,6′-DP) and of Aiolos and Ikaros in the human multiple myeloma MM1.S cell line (1 μM of pomalidomide and of F-3,6′-DP) was evaluated by Western blotting with quantification relative to GAPDH expression. Whereas pomalidomide dramatically lowers SALL4, Aiolos, and Ikaros levels, F-3,6′-DP had no significant effect on these cereblon neo-substrates (Figure 8).

**Figure 9:**
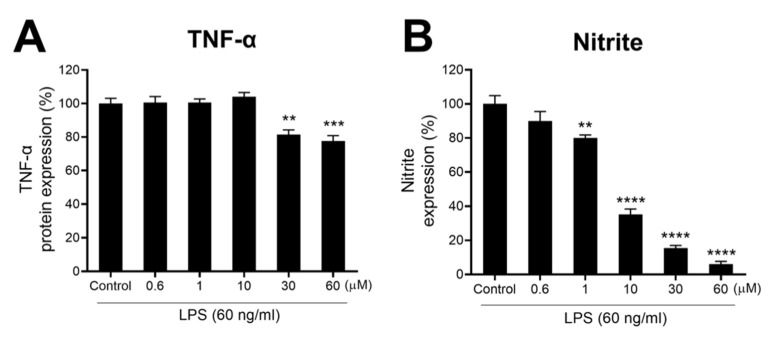
**F-3,6′-DP mitigates LPS-induced inflammation in cultured RAW 264.7 cells.** (**A**,**B**) Cultured RAW 264.7 cells were pretreated with either vehicle or F-3,6′-DP (0.6−60 μM) and challenged with LPS (60 ng/mL) 1 h later. At 24 h following LPS challenge, cellular viability, nitrite (a stable marker of NO generation), and TNF-α levels were quantified. F-3,6′-DP significantly lowered LPS-induced elevations in nitrite and TNF-α levels. **, *p* < 0.01, ***, *p* < 0.001, ****, *p* < 0.0001 vs. the control (LPS + Veh) group.

F-3,6′-DP mitigates LPS-induced inflammation in RAW 264.7 cells. Cultured RAW 264.7 cells were pretreated with either vehicle or F-3,6′-DP (0.6–60 μM) and challenged with LPS (60 ng/mL) 1 h later. At 24 h following LPS exposure, cellular viability, nitrite (a stable marker of NO generation), and TNF-α levels were quantified. F-3,6′-DP was well tolerated and maintained cellular viability at >90% across all concentrations evaluated, and significantly lowered LPS-induced elevations in nitrite and TNF-α levels (Figure 9A,B). 

### 3.3. Potential Differences and Similarities in the Docking Pocket for F-3,6′-DP, Thalidomide and Pomalidomide in Human Cereblon, as Evaluated by Molecular Modeling

**Figure 10:**
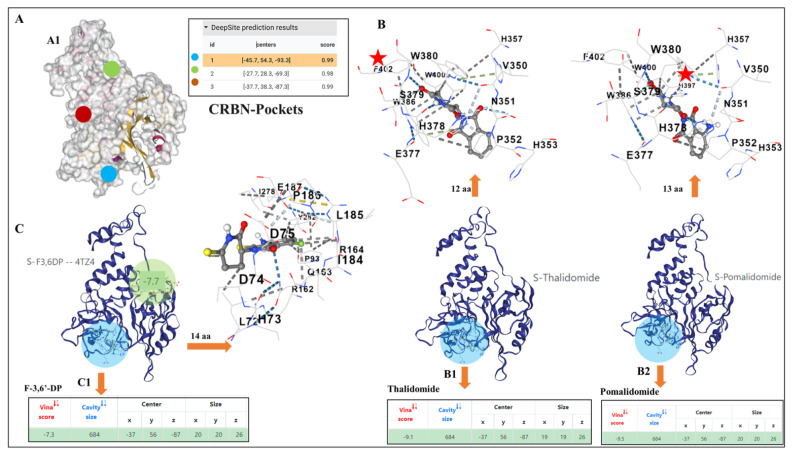
**Drug docking pockets and docking predictions in Cereblon.** Molecular modeling evaluation suggests that cereblon may contain up to three drug docking pockets for IMiDs (**A**,**A1**) (pharmacophore) with their ID and centers marked with a number and colors (A-inset). (**B**) The highest ranked docking pockets for thalidomide (**B**,**B1**-blue circle) and pomalidomide (**B2**-blue circle) are shown as pocket number 1, with the amino acids (aa) that they are predicted to engage within the binding domain (**B**, inset below the models **B1**,**B2**). The top-ranked docking pocket for F-3,6′-DP (**C**) was predicted as pocket number 2 (**C**, green circle), with pocket number 1 (**C**, blue circle) as the lower ranking. The amino acids that F-3,6′-DP is predicted to engage within pocket number 1 are shown (**C**, blue circle), and for the same pocket, thalidomide and pomalidomide were predicted to interact with 12 and 13 amino acids, respectively (**B1**,**B2**, blue circles). Note the highest Vina scores of the S-stereoisomers of pomalidomide −9.5, −9.1 for thalidomide (**B** inset at the bottom) compared to −7.3 for F-3,6′-DP (**C1**, inset at the bottom) within binding pocket number 1. Predicted interactions of the three compounds with proposed binding pocket number 2 are shown in Supplemental Figure S1.

To gain insight into the potential interactions and binding of F-3,6′-DP with cereblon and how this might differ from the conventional IMiDs thalidomide and pomalidomide, molecular modeling studies were performed utilizing the X-ray crystallographic structures of human cereblon (obtained from prior studies involving the IMiD lenalidomide (https//www.rcsb.org/structure/4TZ4, accessed on 27 July 2022) and those of the three compounds evaluated here. These molecular modeling studies open the possibility of human cereblon having more than a single binding domain for IMiDs by predicting that up to 3 potential pharmacophore sites (Figure 10A) may serve as pockets for drug binding. These potential pockets are numbered according to their scores (Figure 10A, inset). The docking prediction suggests that thalidomide and pomalidomide binding/interaction was predicted for pocket number 1 (Figure 10B,B1,B2), which aligns with the pocket identified in X-ray crystallography studies [60,61,75]. Their prediction Vina scores were calculated as −9.1 and −9.5, respectively for the S stereoisomer form of these drugs (Figure 10B1,B2 and insets) with the difference deriving from pomalidomide’s predicted interaction with a greater number of amino acids than thalidomide (Figure 10B,B1,B2 upward directed arrows), associated with the better Vina score for pomalidomide. In contrast, F-3,6′-DP was predicted to engage within pocket number 2, with a Vina score of −7.7 (Figure 10C, small green circle on model, Supplementary Figure S1). Notably, F-3,6′-DP was predicted to also bind in pocket 1, with a lower ranking and prediction Vina score of −7.3 (Figure 10C,C1, blue circle).

These model-associated differences in docking pockets and the nature of bonds that interact with amino acids within the pharmacophore may influence and potentially underpin the differences reflected in the cereblon binding actions on the neo-substrates SALL4, Ikaros, and Aiolos evident in Figure 8B–G.

## 4. Discussion

Within hours after TBI, a variety of cytokines are produced and released to induce local immune cell activation and the attraction of more peripheral immune cells to the injured area, including neutrophils, monocyte infiltration, microglial, and astrocytic activation, as well as reactive oxygen species (ROS) production [16]. Accumulating evidence suggests that neuroinflammation is considered a critical factor among neurodegenerative disorders [16,83,84,85,86,87]. These include epidemiological studies with non-steroidal anti-inflammatory drugs (NSAIDs) against the development of neurodegenerative disorders, such as AD and PD [88,89,90]. However, these studies have largely failed in randomized clinical trials of anti-inflammatory compounds in neurodegenerative diseases [91,92]. Among the many aspects that could account for this failure is that the causes of TBI involve multiple molecular pathological mechanisms, and their temporal profiles and outcomes differ between preclinical animal models and human TBI. Therefore, instead of targeting specific mechanisms as a target-based drug screening strategy, phenotypic screening might be a better approach to determining useful compounds [62,63] that can ameliorate crucial parameters related to TBI, such as behavioral impairments, neuronal death, and microglial phenotypic alteration. Considering that microglial activation appears in many neurodegeneration disorders, such as TBI, and is accompanied by a variety of pro-inflammatory cytokines and chemokines [93,94], such as TNF-α, IL-1β, IL-6, cytokine-suppressive anti-inflammatory drugs (CSAIDs) may prove valuable as a treatment. In this study, we demonstrated that F-3,6′-DP has potent activity in reducing pro-inflammatory cytokines and chemokines, such as TNF-α, KC/GRO (CXCL1), IL-6, and free radicals, such as nitric oxide, ameliorating microglial and astrocytic activation after TBI.

Microglia, a resident glial population in the brain serving as both supporting neuronal health and neuronal destruction during disease, is considered a crucial element in age-related changes [87,95,96]. Age-dependent and senescence-driven deficits in microglial functions, as well as phenotypic alterations, are considered to play important roles during the onset and progression of neurodegenerative disorders [95]. Among the physiological and pathophysiological functions of microglia, the level of TNF-α expressed by microglia is crucial for regulating the homeostatic maintenance of neuronal activities [97,98]. The activation of microglia after TBI usually occurs within 24 h [16,69,99] and extends from weeks to months [100]. In response to various microenvironments, microglia are morphologically and functionally dynamic cells that can change from ramified to completely lacking processes with a larger cell body (amoeboid), usually associated with phagocytic functions [101,102,103]. Early microglial activation after TBI may induce the restoration process of homeostasis in the brain. However, if the activity of microglia is over-excessive and/or remains chronically activated, producing and maintaining pro-inflammatory mediators, this can result in extended brain tissue impairment and the potential for neurodegeneration [102]. The balance of activation status of microglia is relatively dynamic at various timepoints after TBI, but their distribution, morphological changes, and functional phenotypes can provide information to clarify the status of neuroinflammation in preclinical animal models and in humans. The strength of animal models is that they can allow us to manipulate their genetic and pharmacological profiles in order to determine their properties. In this study, we found that the morphological changes in microglia after TBI occur not only in the cortex at the local injury site but also in the thalamus (data not shown). This finding parallels the discovery in clinical studies that found increasing activity of microglia in the thalamus, putamen, and midbrain after TBI [102,104,105,106]. This result may be explained by the thalamocortical radiations, the nerve fibers between the thalamus and cerebral cortex, underlying the sensory or motor functions from the thalamus to many areas of the cortex through relay neurons [107]. It is implied that even subtle neuronal damage, as may occur in normal aging, may trigger morphological changes or the accumulation of secondary microglial activation in areas remote from an injury site. Moreover, we found that the number of microglia can be elevated by TBI specifically on the ipsilateral side; this phenomenon appears not only in the cortex (Figure 6B) but also in the thalamus (data not shown). After F-3,6′-DP treatment, we detected a small reduction in the number of microglia induced by TBI. However, our microglial morphological analysis showed marked differences between the vehicle and drug treatment groups in the TBI mice (Figure 7). This indicates that the amount and morphology of microglia provide different indices of microglia activity during neuroinflammation. 

Gait impairment is a classical marker in clinical TBI populations [4,108,109]. Gait analysis in animal models of TBI is important because most injury models directly affect the motor circuits that control gait function. It is a clinically related symptom that has the potential to translate from “bench to bedside”. Studies have shown that different severity levels of CCI in rodent TBI models can induce both cognitive and motor impairments differently [110,111]. The DigiGait system provides us with detailed gait parameters by using a camera detection system and identifies several gait parameters across animals and trials. In this study, we observed that the majority of gait deficits induced by TBI occurred on the contralateral (left) side, especially in the left hind limb, which parallels the data from previous studies [112,113] and our observation of footfalls during the beam walking test. Notably, craniotomy itself can induce certain changes in gait, based on a previous study [113], and hence the sham group we used here only received skin incisions without craniotomy. Indeed, it is also debatable whether receiving craniotomy is an ideal control for the CCI model of TBI [114]. We observed a subtle elevation of astrocytes and microglia at the surface of the cortex around the craniotomy site (data not shown), which coincides with a previous study [115]. The mechanism(s) of gait impairment caused by TBI remain to be clarified. However, neuronal networks in the spinal cord directly involve the movements of limbs and coordination of gait during locomotion [116] and cortical injuries after CCI can induce microglial activation along the corticospinal tract [117], which provides a potential pathway for understanding the mechanisms behind gait impairments after TBI. 

The temporal difference between the motor behavioral changes after CCI and the histochemical and biochemical findings deserves further comment. Although the changes in motor behavior are clear-cut at one week after CCI, they are largely compensated for at 2 weeks. However, the histochemical and biochemical changes in microglia and cytokines/chemokines are still apparent after this time point [102,104,105,106]. There are documented motor compensatory pathways, both ipsilateral in the injured hemisphere and contralateral via corpus callosum pathways, as documented in extensive preclinical and clinical studies. Interestingly, a similar temporal disparity is seen in stroke, where there is significant age-related motor recovery despite little or no change in lesion size [70]. Although the analysis is imperfect, we can compare the mouse lifespan of about 2 years with a human lifespan of 70 years. Under these circumstances, 2 weeks in a mouse would be equivalent to 70 weeks in humans. This is a period of time when motor deficiencies in human TBI are often partially resolved, but other clinical problems are still apparent. Another issue could involve the types of motor behaviors analyzed here. Both ambulation on a narrow beam and walking on a treadmill involve balance, as well as simple movement parameters. The clinical literature suggests that whereas simple walking can often recover within months after injury, the recovery of balance, which requires both vestibular and proprioceptive function, is often delayed.

This study demonstrates that the novel thalidomide-like drug F-3,6′-DP mitigates TBI-induced impairments across cellular and animal models and achieves this by quelling neuroinflammation, which can be over-excessive following TBI [69,118,119]. Mechanisms underpinning the anti-inflammatory and wider pharmacological actions, as well as known toxicities of thalidomide/pomalidomide-like drugs have been extensively evaluated [49,50,51], and hence are not documented in detail here. 

Cereblon, originally identified as a gene that, when mutated, results in mild mental retardation [120,121], is also a substrate receptor subunit of a ubiquitin E3 ligase complex with the proteins DNA damage-binding protein-1 (DDB1), Cullin 4 (Cul4A/B), and a regulator of Cullins 1 (RoC1) [59,122]. The cereblon portion of this complex directs endogenous proteins for degradation via a ubiquitin-proteasome pathway in order to optimize systemic and brain function and maintain homeostasis [123]. The binding of thalidomide-like drugs to cereblon can modify the endogenous substrates that are targeted for ubiquitination. This, consequently, can halt the breakdown of select proteins routinely targeted by cereblon, such as MEIS2, involved in development, and, alternatively, by altering substrate recognition (contingent on the ligand structure) induce the ligand-specific proteolysis of neo-substrates, such as the zinc finger proteins Ikaros and Aiolos [120,124,125] and CK-1α, to ultimately cause down-regulation of transcription factors such as interferon regulatory factor 4 (IRF4) and c-Myc. Depletion of Ikaros, Aiolos, and CK1α is particularly toxic for malignant cells in hematological cancers, accounting for FDA approval of thalidomide-like drugs for multiple myeloma [61,126]. SALL4 docks at the same site on the cereblon surface as these other neo-substrates in the presence of thalidomide-like drugs, and its degradation mediates teratogenicity [76,127]. Ito at al. [59,75,120], demonstrating binding of conventional IMiDs to cereblon, brought mechanisms potentially accounting for thalidomide’s anti-proliferative, anti-angiogenic and immunomodulatory activity, as well as teratogenic actions under a common mechanistic construct [126].

In terms of cereblon-mediated biological actions of IMiDs, recent studies involving the humanization of murine cereblon in which a single amino acid within the binding domain for thalidomide was changed in the human cereblon sequence (*Crbn*^I391V^) [128], revealed that this single amino acid difference is responsible for mouse cereblon having resistance to thalidomide-like human drug actions, such as the degradation of Ikaros, Aiolos, CK-1α and other neo-substrates that occurs in humans but not mice [129]. Notably, although there are >20 amino acid differences between mouse and human cereblon, the V388 residue in human cereblon parallels I391V in the mouse. This appears to be associated with the teratogenicity of classical IMiDs, with lenalidomide and thalidomide causing a high rate of fetal loss in cereblon-humanized but not wild-type mice [130]. Concerning anti-inflammatory mechanisms, cereblon has a regulatory role in inflammatory processes initiated via toll-like receptor 4 (TLR4) stimulation, through which the actions of LPS and other potential inflammatory factors are mediated [131]. Cereblon-induced modulation of TNF receptor-associated factor 6 (TRAF6), acting via TAK1, regulates NF-κB activation and the production of pro-inflammatory cytokines that include IL-6 and IL-1β. Additionally, cereblon can suppress LPS-induced inflammatory responses via several mechanisms, including promoting ubiquitination and the degradation of c-Jun [129]. Notably, conventional IMiDs have been described as inducing anti-inflammatory actions in both wild-type and cereblon-humanized mice [128,130]. The present communication and our prior studies [73,132] further support this separation of pathways underlying neuroinflammation, with some pathways being mediated via cereblon and others being independent of it. In the current study, we show that the novel thalidomide-like compound F-3,6′-DP effectively ameliorates systemic and brain inflammation, but although it binds to the key protein cereblon, it does not efficiently trigger the degradation of transcription factors (SALL4, Ikaros, and Aiolos) associated with the teratogenic and anti-proliferative responses induced by this drug class, thereby providing a different profile to pomalidomide.

The question therefore arises as to why conventional IMiDs, such as thalidomide and pomalidomide, bind to cereblon and trigger the degradation of the neo-substrates SALL4, Ikaros, and Aiolos, whereas structurally close analogs, such as F-3,6′-DP in the current study and 3,6′-DP in our former studies [73,74] bind cereblon but do not then trigger alike neo-substrate degradation. Molecular modeling studies were thus undertaken to define conceivable factors underpinning the differential cereblon-related actions of F-3,6′-DP versus the clinically approved IMiDs thalidomide and pomalidomide, and to provide new potential directions for future evaluation. These modeling studies suggest that further docking pockets may exist for IMiD-like drugs on the surface of cereblon, in addition to the pocket reported in X-ray crystallography studies of cereblon co-incubated with lenalidomide, thalidomide, and pomalidomide [61,127], which aligns with pocket number 1 in our studies (Figure 10A). Our modeling studies suggest the prospect that the S-stereoisomer of F-3,6′-DP differentially interacts with key amino acids within a separate adjacent pocket (Figure 10C: pocket number 2, green circle), which provided F-3,6′-DP its best ranked binding scores as compared to pocket number 1 (Figure 10C1, blue circle), where it was also predicted to bind with a lower ranking. Such differential binding may support the results of the cereblon/BRD3 binding FRET assay through possible allosteric interactions of binding pocket number 2 with binding pocket number 1, but not support the interaction of SALL4, Ikaros, and Aiolos with F-3,6′-DP bound within pocket number 2. Binding and ensuing neo-substrate degradation are known to occur with clinically approved conventional IMiDs bound in pocket number 1 (Figure 10B,B1,B2) and, moreover, provide an avenue for future research. Likewise, it would be interesting to evaluate whether similar differences exist in the interaction of the IMiDs with known inflammatory mediators involved in TBI and are known to chronically persist in neurodegenerative disorders in order to unravel the potential mechanism of actions of conventional, as well as new generation IMiDs. 

Molecular modeling has its own limitations, as it exploits the rules of quantum and/or classical physics when attempting to simulate and examine interacting molecular entities. Protein–drug binding is a highly challenging molecular modeling puzzle, with thousands of protein atoms/amino acids interacting with each other and with those of the drug of interest via complex molecular mechanisms in the presence of water. The protein and drug have flexibility and can thus accommodate multiple configurations as they interact, as numerous bonds within both the protein and drug can potentially twist, bend, and vibrate. Drug ligands, and particularly thalidomide analogs, can be malleable and take on a wide variety of possible 3-dimensional configurations, especially when different potential ionization states, tautomers, and stereoisomers may exist. Likewise, prior X-ray crystallography studies of cereblon co-incubated with thalidomide-like drugs [60,61,75,127] have their advantages and caveats, and provide valuable insight into target-drug interactions. Additionally, future studies involving cereblon amino acid mutations that lead to a lack of IMiD action may also prove constructive in understanding how classical and new IMiDs mediate their pharmacological actions. 

In summary, neuroinflammation is a potential target for drug development in TBI and other neurodegenerative disorders. Based on the chemical structure of thalidomide, the IMiD drug class, which includes the clinically available structural analogs pomalidomide and lenalidomide, can reduce TNF-α expression during inflammation and represent a novel treatment strategy for drug repurposing. Unfortunately, these drugs have several adverse effects [50,133]. F-3,6′-DP, as a novel class of IMiDs, demonstrates activities in ameliorating neuroinflammation and behavioral impairments induced by TBI in mice, with differential regulation of key protein levels involved in cereblon-mediated biological actions of the IMiD drug class [61,122,126]. Hence, F-3,6′-DP, as a novel candidate compound, deserves further investigation in drug development for neurodegenerative disorders potentially driven by over-excessive or chronic neuroinflammation. In undertaking this task, future evaluation in female rodents is a requisite in the drug development process, as well as being scientifically important, as there are substantial sex differences in outcomes from TBI. As reviewed by Gupte and colleagues [134], there are differences in the manner in which male and female brains may react to TBI at a cellular and molecular level, with genetics and sex hormones (in particular, estrogen [135]) likely differentially impacting ensuing systemic and neuroinflammation, oxidative stress, excitotoxicity, edema, and other phenomena. There are likely differences in brain repair and recovery processes in relation to involved mechanisms and their time-dependence between males and females. Finally, there are possible sex differences in response to the outcome measures selected for evaluation, in addition to pharmacokinetic gender differences in how a drug is handled [136,137]. Our preliminary data across cellular and animal models of inflammation, together with cereblon and TBI studies, suggest that such future studies are warranted for F-3,6′-DP and similar novel IMiDs.

## 5. Conclusions

F-3,6′-DP can lower the levels of free radicals, such as nitric oxide, in vitro, and pro-inflammatory factors, such as IL-6 and KC/GRO (CXCL1), in vivo. Notably, TNF-α, the key pro-inflammatory cytokine that initiates and drives inflammation, can be efficiently downregulated by F-3,6′-DP. In a classical mouse model of moderate TBI, F-3,6′-DP reduced gait impairment and lesion area and ameliorated the increment of astrocytes, as well as morphological changes in microglia activation state, all established hallmarks of neuroinflammation. F-3,6′-DP’s interactions with cereblon, a primary target of IMiDs, are different from clinically approved members of this drug class and lack action on proteins associated with adverse effects; thus, they may lead to a safer profile.

## Data Availability

Data is contained within the article and Supplementary Material.

## Supplementary Materials

The following supporting information can be downloaded at: https://www.mdpi.com/article/10.3390/biomedicines10102449/s1, Figure S1: Drug docking pockets and docking predictions in Cereblon pocket number 2. Molecular modeling evaluation suggests that cereblon conceivably possesses up to three top ranked drug docking pockets for IMiDs (A) predicted cereblon binding pockets with their ID and centers marked with a number and color. (B) The highest ranked predicted cereblon docking pocket for S enantiomeric F-3,6′-DP (green circle: pocket number 2) and associated computed Vina scores and cavity sizes. The two highest top ranked computed cereblon binding pockets for the S enantiomeric forms of thalidomide and pomalidomide are shown (C).
