# Supplementary material for: Activity of a Novel Anti-Inflammatory Agent F-3,6′-dithiopomalidomide as a Treatment for Traumatic Brain Injury
Source: Biomedicines. 2022 Sep 30;10(10):2449. doi: 10.3390/biomedicines10102449 (PMC9598880; doi:10.3390/biomedicines10102449)
Supplement: Supplementary file 1 [file biomedicines-10-02449-s001.zip › biomedicines-1900631-supplementary.pdf]

Activity of a Novel Anti-Inflammatory Agent F-3,6′-dithiopomalidomide as a Treatment for Traumatic Brain Injury

Shih Chang Hsueh [1], Michael T Scerba [1], David Tweedie [1], Daniela Lecca [1], Dong Seok Kim [2,3], Abdul Mannan Baig [4], Yu Kyung Kim [3], Inho Hwang [3], Sun Kim [3], Warren R Selman [5], Barry J Hoffer [5] and Nigel H Greig [1,*]

[1] Drug Design & Development Section, Translational Gerontology Branch, Intramural Research Program National Institute on Aging, NIH, Baltimore, MD 21224, USA
[2] AevisBio, Inc., Gaithersburg, MD 20878, USA
[3] Aevis Bio, Inc., Daejeon 34141, Korea
[4] Department of Biological and Biomedical Sciences, Aga Khan University, Karachi 74800, Pakistan
[5] Department of Neurological Surgery, Case Western Reserve University and University Hospitals, Cleveland, OH 44106, USA
* Correspondence: greign@grc.nia.nih.gov

Supplementary File. 1

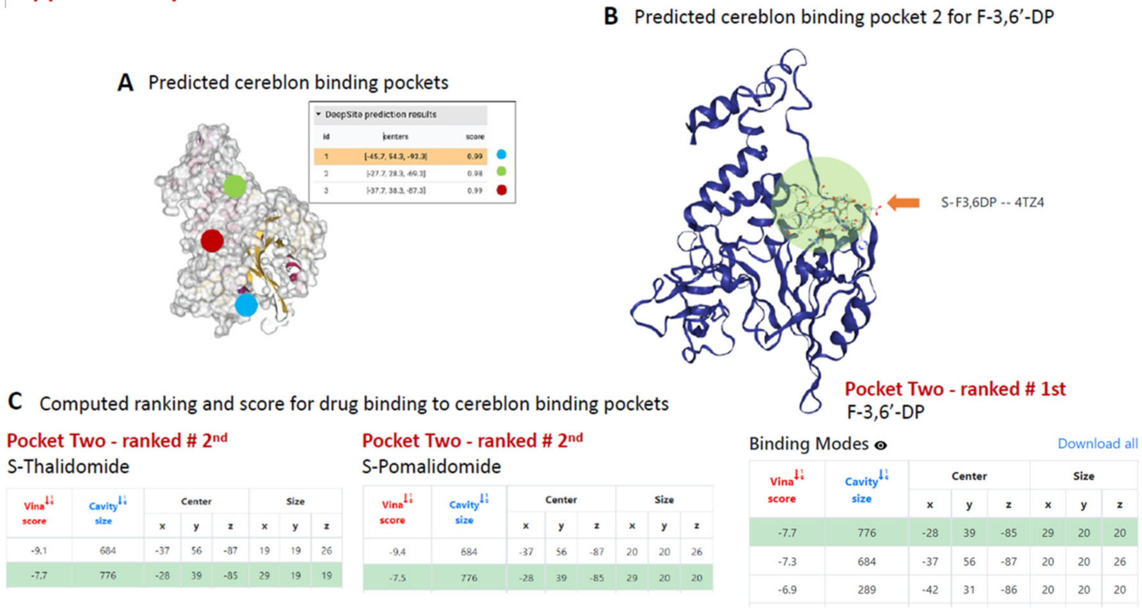

Figure S1. Drug docking pockets and docking predictions in Cereblon pocket number 2. Molecular modeling evaluation suggests that cereblon conceivably possesses up to three top ranked drug docking pockets for IMiDs (A) predicted cereblon binding pockets with their ID and centers marked with a number and color. (B) The highest ranked predicted cereblon docking pocket for S enantiomeric F-3,6′-DP (green circle: pocket number 2) and associated computed Vina scores and cavity sizes. The two highest top ranked computed cereblon binding pockets for the S enantiomeric forms of thalidomide and pomalidomide are shown (C).
